# A Systematic Review of Economic Evaluations of Treatments for Borderline Personality Disorder

**DOI:** 10.1371/journal.pone.0107748

**Published:** 2014-09-29

**Authors:** Christian Brettschneider, Steffi Riedel-Heller, Hans-Helmut König

**Affiliations:** 1 Department of Health Economics and Health Services Research, Hamburg Center for Health Economics, University Medical Center Hamburg-Eppendorf, Hamburg, Germany; 2 Institute of Social Medicine, Occupational Health and Public Health, University of Leipzig, Leipzig, Germany; Tulane University School of Public Health and Tropical Medicine, United States of America

## Abstract

**Purpose:**

The borderline personality disorder is a common mental disorder. It is frequently associated with various mental co-morbidities and a fundamental loss of functioning. The borderline personality disorder causes high costs to society. The aim of this study was to perform a systematic literature review of existing economic evaluations of treatments for borderline personality disorder.

**Materials and Methods:**

We performed a systematic literature search in MEDLINE, EMBASE, PsycINFO and NHSEED for partial and full economic evaluations regarding borderline personality disorder. Reported cost data were inflated to the year 2012 and converted into US-$ using purchasing power parities to allow for comparability. Quality assessment of the studies was performed by means of the Consensus on Health Economic Criteria checklist, a checklist developed by a Delphi method in cooperation with 23 international experts.

**Results:**

We identified 6 partial and 9 full economic evaluations. The methodical quality was moderate (fulfilled quality criteria: 79.2% [SD: 15.4%] in partial economic evaluations, 77.3% [SD: 8.5%] in full economic evaluations). Most evaluations analysed psychotherapeutic interventions. Although ambiguous, most evidence exists on dialectical-behavioural therapy. Cognitive behavioural therapy and schema-focused therapy are cost-saving. Evidence on other interventions is scarce.

**Conclusion:**

The economic evidence is not sufficient to draw robust conclusions for all treatments. It is possible that some treatments are cost-effective. Most evidence exists on dialectical-behavioural therapy. Yet, it is ambiguous. Further research concerning the cost-effectiveness of treatments is necessary as well as the identification of relevant cost categories and the validation of effect measures.

## Introduction

According to the International Classification of Diseases 10^th^ revision (ICD-10) the borderline personality disorder (BPD) is an emotionally unstable personality disorder (F60.3). The ICD-10 defines BPD by the following characteristics: emotional instability, lack of impulse control, disturbances in self-image, aims and internal preferences, chronic feelings of emptiness, intense and unstable interpersonal relationships, self-destructive behaviour, including suicide gestures and attempts [1].

BPD is a common disorder. In two epidemiological studies based on US-American samples the point-prevalence and lifetime prevalence of BPD was 1.6% and 5.9% respectively [2], [3].

BPD is strongly associated with Axis-I disorders. 84.5% of BPD patients are suffering from a 12-month co-morbid Axis-I disorder [2]. Consequences of BPD are severe impairments of social and vocational functioning which precludes nearly half of the BPD patients from recovery [4]. Hence BPD has a substantial impact on society.

Related to the effectiveness of psychological treatments for BPD a recent review of the Cochrane Collaboration concluded that psychotherapy (e.g. dialectical behaviour therapy (DBT), mentalization-based treatment in a partial hospitalisation (MBT), transference-focused therapy (TFP), cognitive behavioural therapy (CBT), dynamic deconstructive psychotherapy (DDP), interpersonal psychotherapy (IPT)) plays an important role in the therapy of BPD. However, the evidence base is not very robust [5].

There are only few studies on the economic burden of BPD. Jerschcke et al [6] reported annual costs for medical care (direct costs) of more than 18,000 US-$ per patient (purchasing power parities) for a German setting. More than 90% of these costs were caused by inpatient care. Van Asselt et al [7] assessed direct costs and productivity losses (indirect costs) in a Dutch population. They found total annual costs of more than 23,000 US-$ (purchasing power parities). These costs were caused by medical care and productivity losses in similar proportions. In another cost-of-illness study Goodman et al [8] found that the kind of co-morbidity has a massive influence on costs of BPD. A co-morbid conduct disorder raised the annual costs of BPD by nearly 50,000 US-$, for example.

In light of this medical and economic burden the necessity to identify effective and efficient treatments is evident.

To analyze the costs and the efficiency of treatments partial and full economic evaluations are the methods of choice. In partial economic evaluations only the costs of at least two alternatives are compared. This kind of evaluation is also called cost analysis [9]. For the calculation of costs different perspectives can be employed. The most frequent perspectives are the perspective of the society and of a third party payer. Two categories of cost can be distinguished: direct and indirect costs. Direct costs arise directly from medical care. This includes the costs for inpatient and outpatient care, rehabilitation, drugs, or emergency room treatments. Indirect costs are defined as the loss of productivity resulting from a disease. This loss arises from reduced productivity at work, sick leave, early retirement or mortality.

A full economic evaluation does not only compare costs of at least two alternatives but also their effects [9]. Effects can be measured in natural units (life years gained, parasuicide events avoided), artificial units (quality-adjusted life years [QALY] or disability-adjusted life years [DALY]) or monetary units measured by techniques like willingness-to-pay experiments. Depending on the effect measure employed full economic evaluations are called cost-effectiveness analyses (natural units), cost-utility analyses (utility measures) or cost-benefit analyses (outcomes valued monetarily).

There are different approaches to perform a partial or a full economic evaluation. The first approach uses primary data collected along a clinical trial evaluating the clinical effectiveness of the specific treatment. Other approaches are based on secondary data and/or decision analytic modelling, e.g. decision trees or Markov models.

The aim of this study was to perform a systematic literature review of economic evaluations of treatments for BPD.

## Materials and Methods

### Search strategy

We conducted a systematic literature search in MEDLINE, EMBASE, PsycINFO and NHSEED in August 2013 based on the following strategy: (cost OR economic) AND (borderline disorder OR borderline personality OR bpd OR 301.83 OR F60.30 OR F60.31 OR Cluster B). The literature search was not restricted by publication year.

### Inclusion and exclusion criteria

The eligibility of the studies was assessed in two steps. First titles and abstracts were screened. Articles considered as relevant were obtained and the full text was screened. All original studies reporting cost or cost-effectiveness data of BPD were included. Articles were excluded if they

- were conference abstracts, editorials, letters or reviews
- were no economic evaluation
- reported no data from a control group
- presented no data for BPD
- did not document the method of cost assessment
- were not written in English or German.

### Data extraction

The extraction process consisted of three steps. First study characteristics were identified. Afterwards the different cost categories considered in the studies were documented. Finally the costs were extracted. Two transformations of the cost data were performed. First, costs per patient were calculated if cost data related to groups or populations. Subsequently all costs were inflated to the year 2012 and converted into US-$ using purchasing power parities (US-$ PPP) to ensure comparability of the data [10]. Furthermore, incremental cost-effectiveness ratios (ICER) and findings from Cost-Effectiveness-Acceptability-Curves (CEAC) were extracted from full economic evaluations. The ICER is the outcome measure of a full economic evaluation and defined as the ratio of the difference in costs and the difference in effects of the treatment alternatives compared. As the ICER is a point estimate it gives no information about the uncertainty of results. To assess the uncertainty of the results CEAC are employed. The CEAC indicates the probability of cost-effectiveness at a specific willingness-to-pay margin. There are different rules of thumb to classify incremental cost per QALY ratios. We employed a widely used [11], [12], [13], [14] threshold of 50,000 US-$ PPP per QALY to distinguish cost-effective from economically unfavourable interventions. In the discussion section we additionally use a threshold of 129,090 US-$ PPP per QALY. This represents a recent update of the calculation of the 50,000 US-$ PPP per QALY threshold mentioned above [15]. As there are no accepted threshold values for cost per avoided parasuicide event, cost per recovered patient and cost per percent point reduction of self-harm incidence we abstained from an interpretation. ICER of that kind were reported and results of the CEAC were referred to a willingness-to-pay margin of 0 US-$ PPP per unit of the effect measure.

### Quality assessment

We used the quality checklist developed by the Consensus on Health Economic Criteria (CHEC) project [16]. This checklist was prepared using a Delphi method (three Delphi rounds; 23 international experts). It comprehends 19 criteria. The results of the quality assessment are displayed as percentage of fulfilled criteria.

## Results

### Study pool

**Figure 1:**
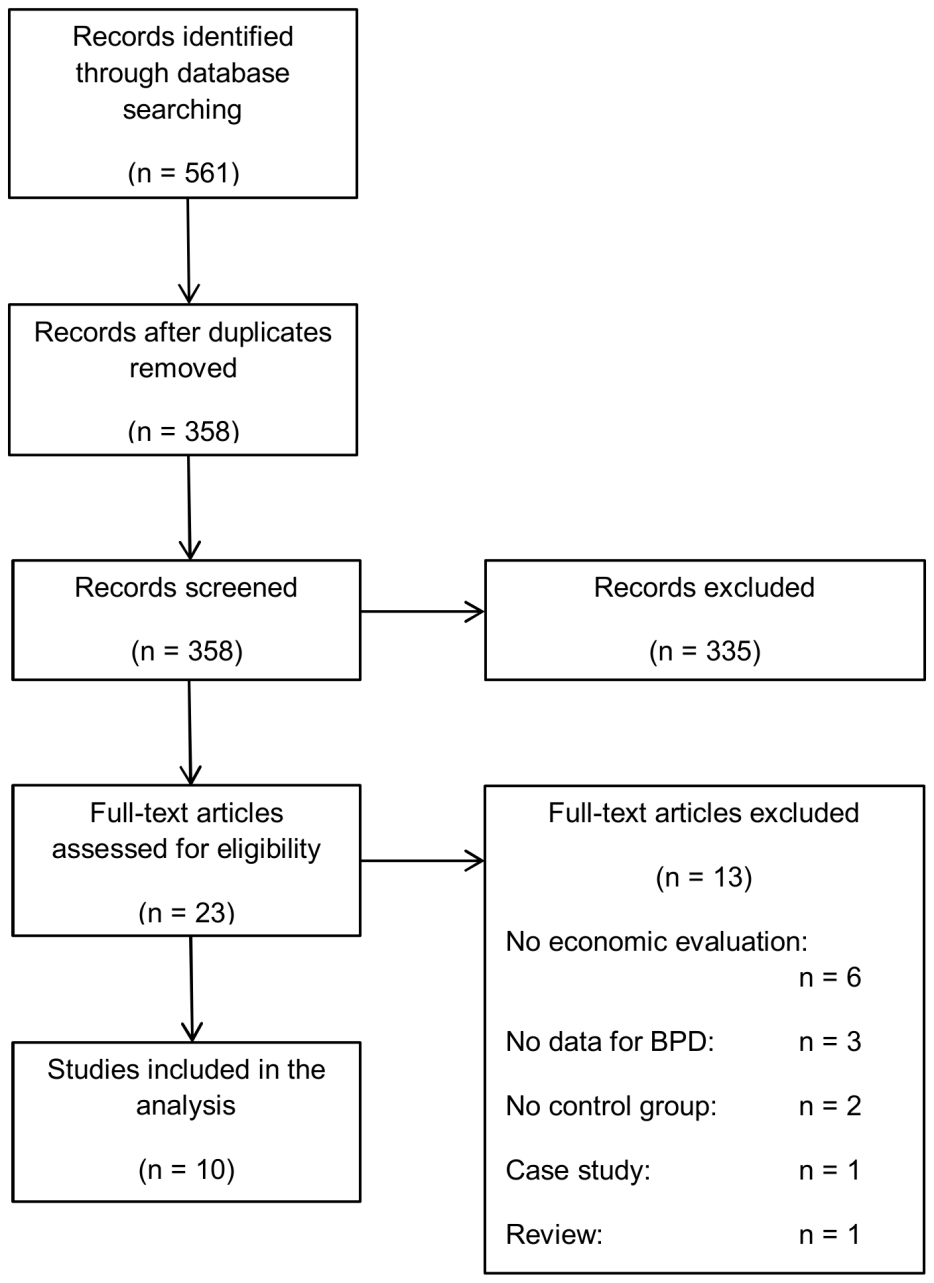
Prisma Flowchart.

The results of the systematic literature search are presented in figure 1. The systematic literature search retrieved 561 results. 159 articles derived from MEDLINE, 235 from EMBASE, 148 from PsycINFO and 19 from NHSEED. 203 articles were duplicates and were removed. After title and abstract screening 335 articles were excluded. Full text screening was performed for the remaining 23 articles. 13 articles were excluded of which six were not economic evaluations, three documents did not report data for BPD, two did not incorporate a control group, one was a case study and one was a review. Finally ten articles were considered in this review. As one of the articles was a HTA report performing six full economic evaluations based on six single RCT [17], this review is based on 15 evaluations (table 1). Nine evaluations were full economic evaluations [18], [19], [20], [21], [22], [23], [24], [25], [26] and six were partial economic evaluations [27], [28], [29], [30], [31], [32]. Nine evaluations were based on clinical trials [24], [25], [26], [27], [28], [29], [30], [31], [32], six on decision analytic models[18], [19], [20], [21], [22], [23]. All modelling studies were full economic evaluations. Eleven evaluations were conducted in the United Kingdom, two in the Netherlands and one each in Australia and Switzerland. The least recent evaluation dated back to 2003, the most recent to 2013. The majority of evaluations employed the societal perspective. The time horizon of the full economic evaluations ranged from one to four years, the time horizon of the partial economic evaluations from three months to six years. Most evaluations included less than 100 patients (n = 11), some even less than 50 (n = 5). In all evaluations the majority of patients was female, in ten evaluations the proportion of female patients was larger than 80%. The mean age of populations ranged from 22 years to 37 years.

**Table 1:** Characteristics of partial and full economic evaluations.

	Study	Country	Study type	Sample size	Perspective	Time horizon	Mean age	Proportion of female patients
Partial economic evaluations	Bateman (2003) [27]	UK	RCT	41	Payer	3 years	31.8	58%
	Van Asselt (2008b) [28]	NL	RCT	86	Society	5 years	30.6	93%
	Davidson (2010) [29]	UK	RCT	106	Society	6 years	31.9	84%
	Pasieczny (2011) [30]	AUS	RCT	90	Society	6 months	33.6	93%
	Berrino (2011) [31]	CH	Controlled study	200	n.a.	3 months	32.1	85%
	Borschmann (2013) [32]	UK	RCT	88	Payer	6 months	35.8	81%
Full economic evaluations	Brazier (2006) [17] based on Turner (2000) [18]	UK	Model	24	Society	1 year	22	79%
	Brazier (2006) [17] based on Linehan (1991) [19]	UK	Model	44	Society	1 year	n.a.	n.a.
	Brazier (2006) [17] based on van den Bosch (2002) [20]	UK	Model	58	Society	1 year	37.5	100%
	Brazier (2006) [17] based on Koons (2001) [21]	UK	Model	28	Society	1 year	35	100%
	Brazier (2006) [17] based on Bateman (1999) [22]	UK	Model	38	Society	1 year	31.8	58%
	Brazier (2006) [17] based on Tyrer (2003) [23]	UK	Model	480	Society	1 year	32	68%
	Palmer (2006) [24]	UK	RCT	106	Society	2 years	31.9	84%
	Van Asselt (2008a) [25]	NL	RCT	86	Society	4 years	30.6	93%
	Priebe (2012) [26]	UK	RCT	80	n.a.	1 year	32.2	88%

*AUS: Australia; CH: Switzerland; NL: The Netherlands; RCT: Randomized Controlled Trial UK: United Kingdom.*

### Methodological quality of identified studies

The results of the assessment of methodological quality are presented in table 2. The partial economic evaluations fulfilled 71.1% (SD: 16.9%) of the quality criteria, the full economic evaluation 78.9% (SD: 5.3%) on average.

**Table 2:** Results of the assessment methodological quality.

	Partial economic evaluations: Cost analyses						Full economic evaluations: CEA, CUA								
Criteria/Author	Bateman (2003) [27]	van Asselt (2008a) [28]	Davidson (2010) [29]	Pasieczny (2011) [30]	Berrino (2011) [31]	Borschmann (2013) [32]	Brazier (2006a) [18]	Brazier (2006b) [19]	Brazier (2006c) [20]	Brazier (2006d) [21]	Brazier (2006e) [22]	Brazier (2006f) [23]	Palmer (2006) [24]	van Asselt (2008b) [25]	Priebe (2012) [26]
Study population clearly described	+	+	+	+	+	+				+	+	+		+	+
Competing alternatives clearly described	+	+	+	+	+	+	+	+	+	+	+	+	+	+	+
Well-defined research question in answerable form	+	+	+	+	+	+	+	+	+	+	+	+	+	+	+
Economic study design appropriate	+	+	+			+	+	+	+	+	+	+	+	+	+
Time horizon appropriate	+	+	+		+	+	+	+	+		+	+	+	+	+
Perspective chosen appropriate	+	+	+	+		+	+	+	+	+	+	+	+	+	
All important and relevant costs identified	+	+				+								+	+
All costs measured appropriately in physical units	+	+	+	+	+	+			+	+			+	+	+
Costs valued appropriately	+	+	+	+	+	+	+	+	+	+	+	+	+	+	+
All important and relevant outcomes identified							+				+	+	+	+	
All outcomes measured appropriately								+	+	+		+	+	+	+
Outcomes valued appropriately							+	+	+	+	+	+	+	+	+
Incremental cost-effectiveness analysis performed							+	+	+	+	+	+	+	+	+
All future costs and outcomes discounted appropriately		+		+	+	+	+	+	+	+	+	+	+	+	+
Sensitivity analysis performed for all important variables whose values are uncertain		+				+	+	+	+	+	+	+		+	
Conclusions follow from the data reported	+	+	+	+	+	+	+	+	+	+	+	+	+	+	+
Generalizability of results to other settings and patient/client groups discussed	+	+					+	+	+	+	+	+			+
Article indicates that there was no potential conflict of interest		+	+			+	+	+	+	+	+	+	+	+	+
Ethical and distributional issues discussed appropriately															
Proportion of criteria fulfilled by the study	73%	93%	67%	53%	53%	87%	74%	74%	78%	78%	78%	84%	74%	89%	78%

*+  =  criterion fulfilled; blank  =  criterion not fulfilled; CEA  =  Cost-effectiveness analysis; CUA: Cost-utility analysis.*

There are criteria which were not fulfilled by some evaluations and could have an influence on the validity of the findings of this review. Firstly, ten analyses did not identify all important and relevant costs [18], [19], [20], [21], [22], [23], [24], [29], [30], [31]. These studies chose a societal perspective and did not consider indirect cost although their study populations were between 22 and 35 years old. Secondly, discounting was not performed in two evaluations, although the time horizon exceeded one year [27], [29]. Thirdly, four studies did not consider all important outcomes [19], [20], [21], [26]. These studies measured parasuicide events or acts of self-harm and no life-years or QALY. ICER based on cost per avoided parasuicide events or acts of self-harm are difficult to interpret. Fourthly, no study discussed ethical or distributional issues. However, the influence of this shortcoming on the interpretation of the study results is small as it does not decrease the quality of measurement and calculation. Information on ethical and distributional issues is important for policy makers and decision makers which is not the aim of this review.

### Cost categories

Table 3 shows the cost categories considered by the partial and full economic evaluations. Direct costs for inpatient (general and psychiatric hospital services) and outpatient treatment were assessed by most evaluations. One exception was the partial economic evaluation by Berrino et al [31] which only measured costs of inpatient care. Van Asselt et al [28] focussed exclusively on indirect costs. However, this is not a methodological shortcoming because this partial economic evaluation assessed the influence of different approaches to the calculation of productivity losses and was based on the same study as the full economic evaluation by van Asselt et al which reported inpatient and outpatient cost. Four full economic evaluations reported in the HTA report by Brazier et al [17] did not report costs for inpatient and outpatient care either. These evaluations are solely based on costs for “further resource use” which was calculated by a regression model with length of stay and number of parasuicide events as independent variables and total costs as dependent variable.

**Table 3:** Cost categories considered in partial and full economic evaluations.

	Study	Model based on…	Direct costs													Indirect cost		
			Out-patient care	Inpatient care	Partial hospital	Emergency Units	Medication	Community care	Alternative therapies	Informal care	Assisted living facility	Out of pocket	Super-vision	Criminal justice services	Further resource use*	Sickness absence	Early retirement	Mortality
Partial Evaluations	Bateman (2003) [27]		+	+	+	+	+											
	Van Asselt (2008a) [28]															+	+	
	Davidson (2010) [29]		+	+					+		+			+				
	Pasieczny (2011) [30]		+	+		+												
	Berrino (2011) [31]			+														
	Borschmann (2013) [32]		+	+		+		+										
Full Evaluations	Brazier (2006a) [17]	Turner (2000) [18]											+		+			
	Brazier (2006b) [17]	Linehan (1991) [19]	+	+		+												
	Brazier (2006c) [17]	Van den Bosch (2002) [20]											+		+			
	Brazier (2006d) [17]	Koons (2001) [21]					+						+		+			
	Brazier (2006e) [17]	Bateman (1999) [22]	+	+		+	+						+		+			
	Brazier (2006f) [17]	Tyrer (2003) [23]											+		+			
	Palmer (2006) [24]		+	+								+			+			
	Van Asselt (2008b) [25]		+	+			+		+	+		+				+	+	+
	Priebe (2012) [26]		+	+			+	+								+		

*+  =  category considered; blank  =  category not considered; COI  =  Cost-of-illness study; OCS  =  Other cost study; CEA  =  Cost-effectiveness analysis.*

Indirect costs were only measured by three evaluations. Indirect costs caused by sickness absence, early retirement and mortality were assessed by van Asselt et al [25] in their full economic evaluation. In their partial economic evaluation they took sickness absence and early retirement into account [28]. Priebe et al assessed indirect costs caused by sickness absence exclusively [26].

### Measures of effectiveness

Table 4 shows the measures of effectiveness. Four different kinds of measures were employed. Six evaluations used QALY calculated based on the EQ-5D as measure of utility [17], [24], [25]. One evaluation additionally employed the proportion of recovered patients [25]. Recovery was defined as a score below 15 on the Borderline Personality Disorder Severity Index (BPDSI) version IV. Six evaluations employed parasuicide events [17]. A parasuicide can be defined as “any intentional acute self-injurious behaviour with or without suicidal intent, including both suicide attempts and self-mutilate behaviors” [19]. In one evaluation the incidence of self-harm was employed [26]. Self-harm was defined as “any act which (a) the individual performed with the intention of self-harm, and (b) caused tissue damage” [26].

**Table 4:** Description of full economic evaluations, ICER and CEAC.

Study	Model based on…	Alternatives	Effects measured	Direct costs	Indirect costs	Effects	ICER	CEAC:
								#% chance of cost effectiveness
Brazier (2006) [17]	Turner (2000) [18]	DBT	Parasuicide Events	$27,866.17	-	2.92 events	DBT dominates	At 0$/event avoided: 80%%
						12.33 events		
								
		CCT	QALY	$37,144.86	-	0.17 QALY gained	DBT dominates	At 50,000$/QALY: 95%
						0.05 QALY gained		
	Linehan (1991) [19]	DBT	Parasuicide Events	$27,774.13	-	6.82 events	DBT dominates	At 0$/event avoided: 53%
		TAU		$29,910.60	-	33.54 events		
	Van den Bosch (2002) [20]	DBT	Parasuicide Events	$30,852.27	-	16 events	$70.80/event avoided	At 0$/event avoided: 65%
		TAU		$29,570.74	-	34,1 events		
	Koons (2001) [21]	DBT	Parasuicide Events	$41,488.61	-	4 events	$76,325.32/event avoided	At 0$/event avoided: 5%
						4.2 events		
								
		TAU	QALY	$26,223.55	-	0.07 QALY gained	$508,835.49/QALY	At 50,000$/QALY: 5%
						0.04 QALY gained		
	Bateman (1999) [22]	MBT	Parasuicide Events	$32,169.21	-	6.1 events	$66.92/event avoided	At 0$/event avoided: 45%
						17.5 events		
								
		TAU	QALY	$31,406.30	-	0,04 QALY gained	$15,258.09/QALY	At 50,000$/QALY: 65%
						0,01 QALY lossed		
	Tyrer (2003) [23]	MACT	Parasuicide Events	$16,957.24	-	4.9 events	TAU dominates	At 0$/event avoided: 40%
						1.7 events		
								
		TAU	QALY	$13,387.02	-	0.19 QALY gained	$71,612.05/QALY	At 50,000$/QALY: 55%
						0.14 QALY gained		
Palmer (2006) [24]	---	CBT	QALY	$11,315.15	-	1.0633 QALY	$69,986.27/QALY	At 50,000$/QALY: 30%
		TAU		$16,245.68	-	1.2042 QALY		
Van Asselt (2008b) [25]	---	SFT	Recovered patients	$10,750.64	4,222.31	23 patients recovered	SFT dominates	At 0$/recovered patient: 95%
						12 patients recovered		
								
		TFP	QALY	$14,033.74	4,534.32	2.15 QALY	$119,837.06/QALY	At 50.000$/QALY: 75%
						2.27 QALY		
Priebe (2012) [26]	---	DBT	Self-harm incidence	$8,661.38	1,686.57	Incremental Effect: 9% reduction*	$54.85/one percent point reduction of self-harm incidence **	n.a.
		TAU		$5,719.40	1,572.30			

CBT: Cognitive Behavioral Therapy; CCT: Client-Centered Therapy; CEAC: Cost-Effectiveness Acceptability Curve; DBT: Dialectical Behavior Therapy; ICER: Incremental Cost Effectiveness Ratio; MACT: Manual-Assisted Cognitive behavioral Therapy; MBT: Mentalization-Based partial hospitalization; n.a.: not available; QALY: Quality Adjusted Life Years; SFT: Schema-Focused Therapy; TAU: Treatment-As-Usual; TFP: Transference-Focused Psychotherapy; * reduction per 2-month period; **ICER based on direct costs per 2-month period and effects per 2-month period.

### Compared alternatives

Nine full economic evaluations investigated psychotherapeutic interventions (table 4). Five evaluations focused on dialectical behavioural therapy (DBT) in comparison to treatment as usual (TAU) [17], [26] or client centered therapy (CCT) [17]. DBT is derived from the strategies of cognitive-behavioural therapy (CBT) and uses directive, problem-oriented techniques like behavioural skill training, contingency management and exposure to emotional cues as well as supportive techniques like reflection, empathy and acceptance [19].

Mentalization based partial hospitalization (MBT) [17], manual assisted cognitive behavioural therapy (MACT) [17] and CBT [24] were investigated by one evaluation each, using TAU for comparison.

MBT is based on the assumption that BPD results from a failure in mentalization which is the ability to reflect about oneself in relation to others and understand their state of mind [17], [22]. MBT aims at the ability of self-reflection of the patient [17]. MACT is a brief therapy which is cognitively oriented and problem-focussed. Originally, it was developed for patients with multiple suicide attempts [17]. CBT is a psychotherapeutic intervention aiming at the modification of cognitions to influence emotions and behaviour [33].

Finally one evaluation compared schema focused therapy (SFT) with transference focused psychotherapy (TFP) [25]. SFT aims at the correction of dysfunctional schemas which control or rule the patient's life [25]. TFP is based on a negotiated treatment contract between patient and therapist. It aims at the integration of good and bad representation of self and others and at the resolution of fixed, primitive internalised object relations [25]

Four partial economic evaluations investigated psychotherapeutic interventions (table 5). One evaluation compared one DBT with TAU [30], one MBT with TAU [27], one CBT with TAU [29], and one SFT with TFP [28]. The remaining evaluations compared a crisis intervention program to TAU after emergency room care [31] and a combination of a joint crisis plan and TAU to TAU alone [32]. The crisis intervention program consisted of a short-term hospitalization after discharge from the emergency care unit. In the joint crisis plan intervention a meeting between the patient and a care coordinator was conducted to develop strategies to cope with crises.

**Table 5:** Description of partial economic evaluations and cost differences.

Study	Alternatives	Method of indirect cost measurement	Direct costs	Indirect costs	Cost Difference
Bateman (2003) [27]	MBT	n.a.	$9,617.36	—	$-5,041.18
	TAU		$14,658.53	—	
van Asselt (2008a) [28]	SFT	lim. HCA	—	$2,130.20	$-416.98
			—	$2,547.18	
		ext. HCA	—	$9,027.77	$442.68
	TFP		—	$8,585.09	
		FCA	—	$979.60	$-430.54
			—	$1,410.14	
Davidson (2010) [29]	CBT	n.a	$1,697.64	—	$-3,135.13
	TAU		$4,832.77	—	
Pasieczny (2011) [30]	DBT	n.a.	$17,802.69	—	$-8,651.74
	TAU		$26,454.43	—	
Berrino (2011) [31]	Crisis intervention after emergency room care	n.a.	$1,543.42	—	$-4,993.62
	Treatment as usual after emergency room care		$6,537.04	—	
Borschmann (2013) [32]	Joint crisis plans + TAU	n.a.	7,871.92	—	$-479.02
	TAU		8,350.94	—	

AUS: Australia; Ext: Extended; FCA: Friction Cost Approach; HCA: Human Capital Approach; Lim: Limited; MBT: Mentalization-based partial hospitalization; NL: Netherlands; SFT: Schema-Focused Therapy; TAU: Treatment-As-Usual; TFP: Transference-Focused Psychotherapy; SD: Standard Deviation; UK: United Kingdom; USA: United States of America.

### Results of partial economic evaluations

The results of the partial economic evaluations are presented in table 5.

#### DBT vs. TAU

DBT was cost saving compared to TAU [30] in one partial economic evaluation (−8,652 US-$ PPP). TAU consisted of clinical case management (engagement, ongoing assessment, planning, linking with community resources, consultation with carers, assistance expanding social networks, collaboration with medical staff, advocacy, individual counselling, living skills training, psychoeducation, crisis management).

#### MBT vs. TAU

MBT was cost-saving compared to TAU in one partial economic evaluation [27] (−5,041 US-$ PPP). TAU consisted of general psychiatric services (regular psychiatric review when necessary, inpatient admission as appropriate, with discharge to nonpsychoanalytic psychiatric partial hospitalization focusing on problem solving, outpatient and community follow-up, no formal psychotherapy).

#### CBT vs. TAU

CBT was cost saving in comparison to TAU in one partial economic evaluation [29] (3,135 US-$ PPP). TAU consisted of all treatment options offered by the British National Health Service (NHS). Patients were unlikely to receive CBT as this was a new treatment [34].

#### SFT vs. TFP

In one partial economic evaluation measuring only indirect costs of SFT and TFP the results were ambiguous [28]. By using a limited human capital approach estimating the value of lost productivity of patients who were employed at baseline and a friction cost approach estimating the value of lost productivity until the worker is replaced, SFT was cost saving in comparison to TFP (−417 US-$ PPP and −431 US-$ PPP). However, by employing an extended human capital approach estimating the value of lost productivity of all patients independent from their work status at baseline, TFP was cost saving in comparison to SFT (443 US-$ PPP).

#### Crisis intervention vs. TAU

Crisis intervention after emergency room care was cost saving in comparison to TAU in one economic evaluation [31] (−4.994 US-$ PPP). Patients in the TAU group were assigned to treatment by an attendant psychiatrist after discharge.

#### Joint crisis plan vs. TAU

A combination of a joint crisis plan and TAU was cost saving in comparison to TAU in one economic evaluation [32] (−479 US-$ PPP). TAU consisted of continued care by the patient's community mental health team after the crisis.

### Results of full economic evaluations

The ICER are presented in table 4.

#### DBT vs. TAU

ICER based on cost per avoided parasuicide event ranged from a dominance (cost savings and better effects) of DBT to 76,000 US-$ PPP per event avoided by DBT [17] (TAU: 60 minutes of individual therapy per week and supportive or psychoeducational groups [21]; alternative referrals, any therapy available in the community [19]; management by original referral source [20]). The probability of dominance (result of the CEAC at a willingness-to-pay for an avoided event of 0 US-$ per QALY) of DBT in these evaluations ranged from 5% to 65%. One evaluation [17] presented ICER in terms of cost per QALY (TAU: 60 minutes of individual therapy per week and supportive or psychoeducational groups [21]). The ICER was higher than 500,000 US-$ PPP per QALY. The probability of cost effectiveness (result of the CEAC at a willingness-to-pay margin of 50,000 US-$ PPP per QALY) was 5%. One evaluation [26] found an ICER of 55 US-$ PPP per one percent point reduction of self-harm incidence (TAU: Referral back to the referrer, encouragement to therapy other than DBT).

#### DBT vs. CCT

DBT dominated CCT in terms of costs per avoided parasuicide event and cost per gained QALY [17] (CCT: supportive therapy based on empathic understanding of the patient's sense of aloneness and support on an individual basis [17]). The probability of cost-effectiveness was 80% to 95%.

#### MBT vs. TAU

The comparison of MBT and TAU [17] led to an ICER of 67 US-$ PPP per avoided parasuicide event (TAU: general psychiatric services, no formal psychotherapy). The probability of domination was 45%. The ICER in terms of cost per QALY was 15,000 US-$ PPP per QALY. The probability of cost effectiveness was 65%.

#### MACT vs. TAU

TAU dominated MACT [17] in terms of cost per avoided parasuicide event (TAU: standard treatment or continuation of existing treatment). The probability of domination of MACT in terms of avoided parasuicide event was only 40%. Furthermore MACT showed an ICER of 72,000 US-$ PPP per gained QALY. The probability of cost effectiveness was 55%.

#### CBT vs. TAU

CBT was cost saving and less effective in comparison to TAU [24] (TAU: all treatments offered by the British NHS). The probability of cost effectiveness was less than 30%.

#### SFT vs. TFP

SFT dominated TFP [25] in terms of the number of recovered patients. This result had a probability of cost-effectiveness of more than 95%. In terms of cost per QALY SFT is cost saving and less effective in comparison to TFP.

## Discussion

The aim of this paper was to review the existing economic evaluations of treatments for BPD. DBT is the best evaluated treatment for BPD (five full economic evaluations; one partial economic evaluation). However, the evidence is ambiguous. Based on a RCT by Turner et al [18], Brazier et al [17] found dominance of DBT and a high probability of cost-effectiveness in comparison to CCT. Comparing DBT to TAU, the four remaining full evaluations found better effects of DBT. However, there were great differences in incremental costs between these studies, ranging from cost savings [17], [30] to large extra costs [17], [26]. Consequently there was a great heterogeneity in the ICER. Brazier et al [17], who performed three of these full evaluations, discussed this heterogeneity in their HTA report. They identified two shortcomings with respect to the estimation of costs. The first shortcoming results from the transfer of data from an US-American RCT to a British setting in one study [19]. As the provision of health care services in the UK differs from the US this approach leads to an underestimation of potential cost savings [17]. The second shortcoming results from the method of cost modelling employed in their studies. Resource use of not reported utilization was estimated by regression models. They concluded that this approach could lead to overestimated costs and consequently to overvalued ICER. According to Brazier et al [17], especially the economic evaluation based on the RCT by Koons et al [21] was heavily influenced by this effect resulting in an extensively overvalued ICER.

MACT was dominated by TAU in terms of avoided parasuicide events and showed an unfavourable ICER in terms of QALY. As the cost estimation of MACT was solely based on Brazier's regression model it is possible that the ICER of MACT was overvalued [17].

MBT was more effective and more costly than TAU [17]. CBT appears to be cost saving [24], [29] and less effective in comparison to TAU [24], [29]. SFT and TFP showed ambiguous results in terms of avoided parasuicide events and QALY [25]. However, SFT seems to be cost-saving [25], [28].

We used a threshold of 50,000 US-$ PPP per QALY. This threshold was based on the cost-effectiveness of dialysis in end-stage renal disease [37]. However, as the practice of dialysis changed over time Lee et al performed a re-evaluation of this threshold and found that it would be 129.090 US-$ PPP per QALY based on current practice [15]. If the new threshold was applied, MACT, CBT and TFP would become cost-effective in terms of cost per QALY although in case of TFP and MACT the comparator was dominant in terms of avoided parasuicide events or recovered patients, respectively.

As evidence for the cost-effectiveness of psychotherapy of BPD is scarce we want to present a further publication which did not meet the inclusion criteria of this review but provides valuable information to complement our findings and to support future research. The study by Soeteman et al evaluated the cost-effectiveness of psychotherapy for cluster B personality disorders [36]. Approximately 78% of included patients suffered from BPD. The authors developed a Markov cohort model based on a non-randomized controlled study. Problems of selection bias were controlled for with the multiple propensity score method. In contrast to the publications in this review focusing on specific psychotherapeutic approaches the publication by Soeteman et al focused on the treatment setting and compared outpatient, day hospital and inpatient psychotherapy. The authors found that depending on the willingness-to-pay threshold outpatient or day hospital therapy are cost-effective alternatives from the payer and the societal perspective. An important point to learn from this study is that the setting of therapy should be considered. Comparisons of different settings for one psychotherapeutic approach were not performed by other studies included in this review.

Although we identified 15 partial or full economic evaluations in total, it is difficult to draw general conclusions from the existing economic evidence. The results of this review must be viewed with caution and should be regarded as preliminary. There are various reasons for this. Firstly, there are several different treatment options for BPD, and we identified - at best - only one full economic evaluation and one partial economic evaluation for most of these treatments. This means that the amount of evidence per treatment option is very limited. Secondly, the reviewed economic evaluations used different comparators which make comparisons between treatments options difficult. Thirdly, effect measures employed in the economic evaluations varied and were sometimes difficult to interpret. While there are different rules of thumb to interpret or classify incremental cost per QALY ratios, there are no useful threshold values for incremental costs per unit of other effect measures such as avoided parasuicide events or recovered patients. Fourthly, even if studies apparently employed identical effect measures they turned out to differ substantially when regarded more carefully: The six studies that employed avoided parasuicide events for measuring effects used three different definitions for this effect measure. Moreover, of the six studies that employed QALY based on the EQ-5D, three studies did not apply the EQ-5D in a direct patient interview but used a mapping algorithm to transform scores from other instruments into EQ-5D values. Yet, the validity of mapping algorithms is inferior compared to the direct application of the EQ-5D [35]. Additionally, the validity of the employed mapping algorithm was further limited as it was neither developed in a BPD population nor based on a BPD-specific instrument [36]. Fifthly, it is arguable whether the EQ-5D is valid in BPD patients. Van Asselt et al [25] found that ICER calculated based on recovered patients (defined by BPDSI score) and QALY (based on the EQ-5D index) led to contradictory results. This means that the improvement of symptoms of BPD as measured by the validated BPDSI [37] had no positive effect on HRQL as measured by the EQ-5D. In contrast, Soeteman et al found in their cost-effectiveness analysis of psychotherapy for cluster B personality disorders –which incorporated a great proportion of BPD patients (78%)- that the EQ-5D is sensitive to changes in the health status of cluster B patients [38]. Based on this one may assume that the EQ-5D is applicable in BPD population. Nevertheless, a validation study is needed which assesses validity, reliability and responsiveness of the EQ-5D in this population to prove the psychometric properties in a formal way.

## Conclusion

The economic evidence is not sufficient to draw robust conclusions. It is possible that some treatments are cost-effective. The evidence on DBT is the most extensive but ambiguous. Further research is needed which avoids the methodological shortcomings of existing studies. The assessment of costs was heterogeneous and partly even biased. Future research should identify relevant cost categories in cost-of-illness studies. These findings should be incorporated in economic evaluations. Moreover, different and partly even inappropriate approaches to effect measurement were employed and unvalidated instruments were used. Validation studies should be performed and instruments should be chosen whose results can be interpreted in an economic context. A consensus on cost and effect measurement is highly needed.

## Supporting Information

Checklist S1 **PRISMA 2009 Checklist.**
(DOC)
